# Primary ovarian carcinoid: Two cases report and review of literature

**DOI:** 10.1097/MD.0000000000021109

**Published:** 2020-10-02

**Authors:** Li-Rong Zhai, Xi-Wen Zhang, Tong Yu, Zhen-De Jiang, Dong-Wei Huang, Yan Jia, Man-Hua Cui

**Affiliations:** a Department of gynecology and Obstetrics; b Department of Orthopaedics; c Department of Pathology, The Second Hospital of Jilin University, Changchun, Jilin Province, China.

**Keywords:** carcinoid, carcinoid syndrome, ovarian carcinoid

## Abstract

**Introduction:**

Carcinoid tumor is one of the most frequent neuroendocrine tumors, and the majority of which are usually observed in the lungs and gastrointestinal tract. The prevalence of ovarian carcinoids is merely 0.1% in ovarian neoplasms and 1% in carcinoid tumors. We described 2 rare cases in our hospital of primary ovarian carcinoid (POC), causing carcinoid syndrome (CS) of the diarrhea, constipation, and carcinoid heart disease. Besides, we also reviewed related literatures about its origin, variant, clinical manifestation, diagnosis methods, pathological features, treatment strategies and prognosis from 2009 to 2019.

**Patient concerns:**

Case 1 was a 61-year-old postmenopausal woman and presented with diarrhea, abdominal pain, enlargement, bloating and dizziness. Case 2 was a 49-year-old patient who complained of constipation, abdominal pain, bloating, and headache.

**Diagnosis:**

Both patients were diagnosed as primary ovarian carcinoid, insular type.

**Interventions:**

Total abdominal hysterectomy (TAH), bilateral salpingo-oophorectomy (BSO), omentectomy, pelvic lymphadenectomy, and appendectomy without chemotherapy were performed in case 1. Cervix resection, right salpingo-oophorectomy, appendectomy, and pelvic lesion resection with chemotherapy was conducted in case 2.

**Outcomes:**

Both patients achieved satisfactory treatment effects. The follow-up period was 18 and 17 months in case 1 and case 2, respectively. Case 1 encountered carcinoid heart disease and received percutaneous transluminal coronary angioplasty (PTCA) postoperatively. Case 2 suffered multiple metastases postoperatively. However, after effective treatment, both patients were in good condition during follow-up duration.

**Conclusion:**

POC is an extraordinarily rare disease, and commonly with a satisfactory outcome. TAH+BSO with or without postoperative chemotherapy has been considered as an acceptable treatment strategy for POC patients.

## 1 Introduction

Neuroendocrine tumor is a kind of epithelial neoplasm which is mainly differentiated from neuroendocrine cell. Most carcinoid tumors usually influence the function of bronchopulmonary and gastrointestinal tract,^[1]^ however, with other locations like gynecologic system far less common.[^2^,^3^,^4^] POC, which is a relatively rare disease, only composes approximately 1% of all carcinoid tumors and less than 0.1% of all ovarian cancers,[^5^,^6^,^7^] was first described by Stewart et al in 1939.^[8]^

Noh et al^[7]^ claimed that POC was classified into trabecular, strumal, mucinous, and insular types, among which the latter is the most prevalent type and the only 1 associated with CS. CS,^[9]^ including diarrhea, constipation, cyanosis, flushing, dyspnea, bronchospasm, and heart disease,^[9]^ could accompany with carcinoid tumor. To the best of our knowledge, POC presented with diarrhea and constipation has rarely been reported. Therefore, we presented 2 POC cases accompanied by diarrhea and constipation respectively. In addition, we also reviewed related literatures about its origin, variant, clinical manifestation, diagnosis methods, pathological features, treatment strategies, and prognosis from 2009 to 2019.

## 2 Ethic

This case report was approved by the institutional review board of the Second Hospital of Jilin University. Informed written consent was obtained from the patient for the publication of this case report and accompanying images.

## 3 Methods

We reported 2 cases of POC with different clinical symptoms and reviewed relevant literatures in PubMed, Web of Science Core Collection, Library of Congress and LISTA from 2009 to 2019 (Table 1).

**Table 1:**
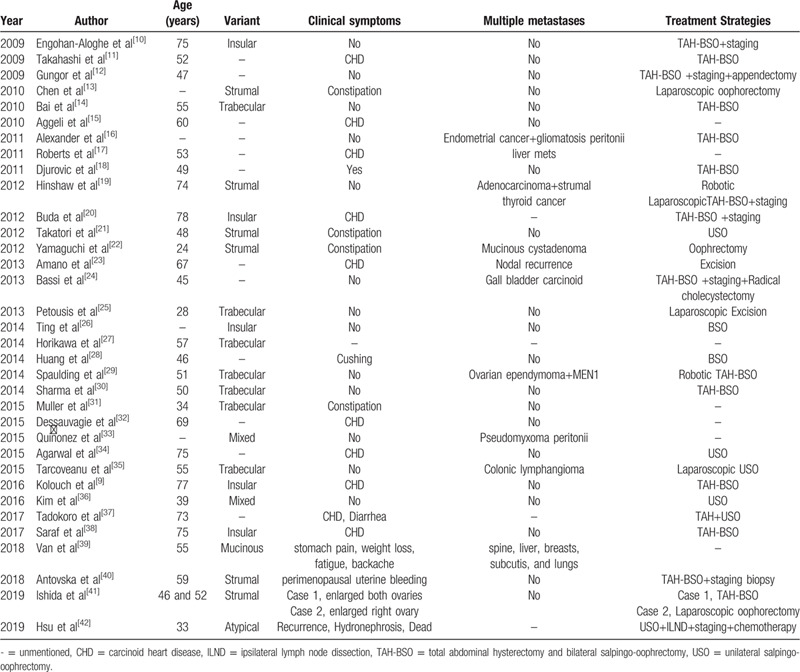
we collected cases of ovarian carcinoid from 2009 to 2019 with their disease characteristics.

## 4 Case 1

### 4.1 Basic characteristics of patient

A 61-year-old postmenopausal woman presented with diarrhea, abdominal pain, enlargement, bloating, and dizziness came to our outpatient. A huge mass about 13.0 cm × 10.0 cm in size was palpated in the right abdomen.

### 4.2 Clinical examination

**Figure 1:**
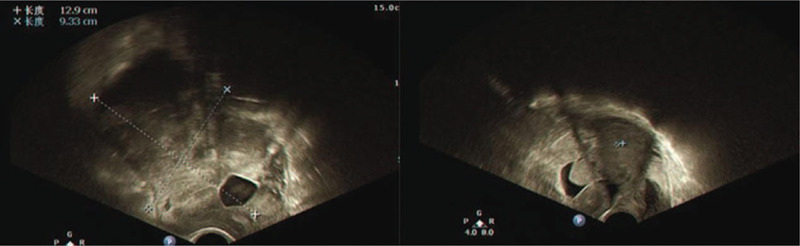
Ultrasound showed that in the right ovarian there was a mass consist of cystic and solid tissue with irregular shape, blurred boundaries and abundant blood flow signal inside.

**Figure 2:**
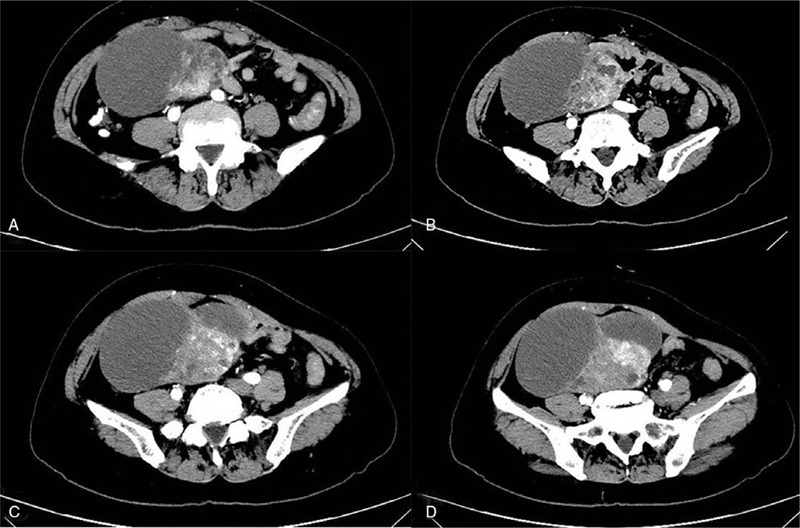
CT scan showed pelvic space-occupying lesions, which may be malignant ovarian tumors.

Ultrasound result (Fig. 1) showed that in the right adnexa region there was a mass consisting of cystic and solid tissues with irregular shape, blurred boundary, and abundant blood flow signal. Blood pressure was 177/101 mm Hg, an abnormal T wave with ST slightly shifted down was showed in electrocardiogram (ECG) and decreased function of the left ventricular was detected by echocardiograph. The results of tumor markers were normal. CT scan (Fig. 2) showed pelvic space-occupying lesions indicating malignant ovarian tumors.

### 4.3 Treatment strategies

**Figure 3:**
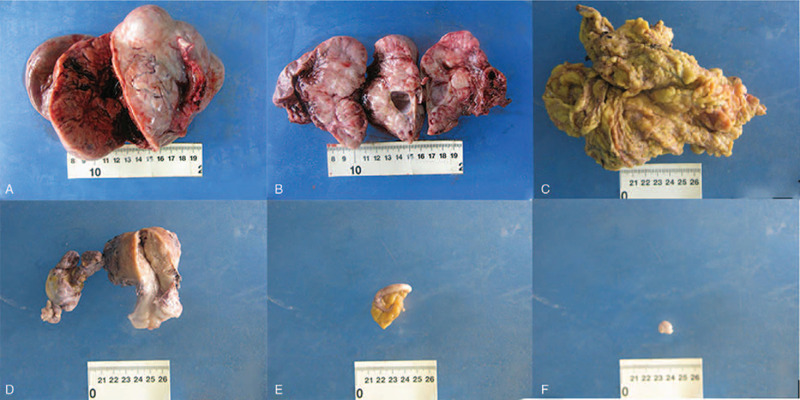
Figure 3 (A-F) showed the tissues resected by laparotomy in case 1.

Laparotomy, including TAH, BSO, omentectomy, pelvic lymphadenectomy, and appendectomy, was performed (Fig. 3). There was no tumor metastasis or invasion, thus, chemotherapy was not carried out postoperatively.

### 4.4 Pathological characteristics

**Figure 4:**
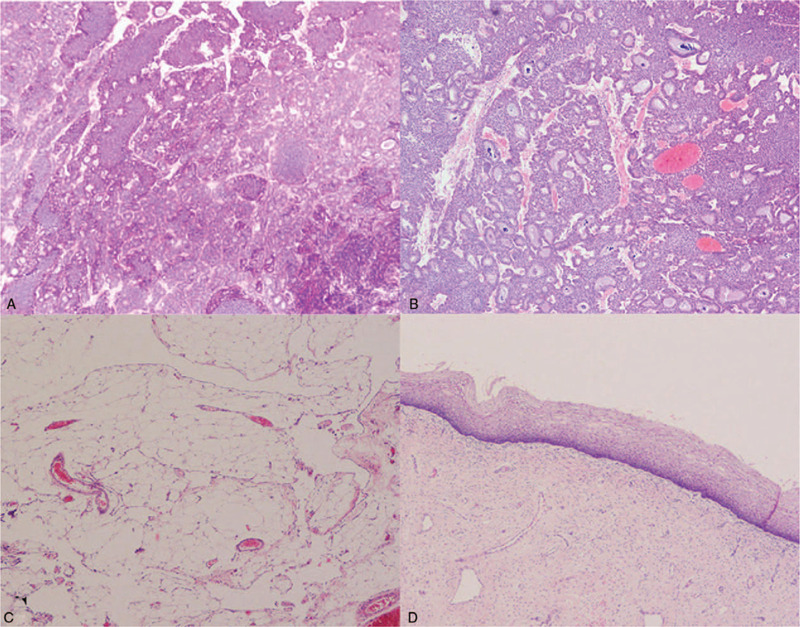
Pathological results showed ovary carcinoid, insular type.

The result of pathological examination (Fig. 4) was ovary carcinoid, insular type. Immunohistochemical (IHC) results were shown as follows: CK (AE1/AE3), synaptophysin (Syn), chromogranin (CgA), CD56, CK20, CDX2, and SATB2 were positive. Vimentin, calretinin, CK7, p63, GATA3, α-inhibin, TTF-1, and PAX-8 were negative. Besides, the positive index of Ki67 was 5%. The patient was diagnosed with POC, stage IAI (according to the 2014 The International Federation of Gynecology and Obstetrics (FIGO) staging classification for ovarian cancer^[43]^).

### 4.5 Clinical outcomes and follow up

The follow up period of this patient lasted 18 months. The patient received PTCA for treatment of myocardial infarction 6 months postoperatively. The general condition recovered to good at the 11 months follow-up visit. No symptoms of discomfort, including abdominal pain, diarrhea, and hypertension, were observed at the last follow-up.

## 5 Case 2

### 5.1 Basic characteristics of patient

A 49-year-old woman, who received transabdominal subtotal hysterectomy and bilateral ovarian partial resection for leiomyoma and bilateral ovarian masses 7 years ago, complained of constipation, abdominal pain, bloating, and headache. A mass about 7.0 cm × 6.0 cm in size was palpated upon gynecological examination.

### 5.2 Clinical examination

**Figure 5:**
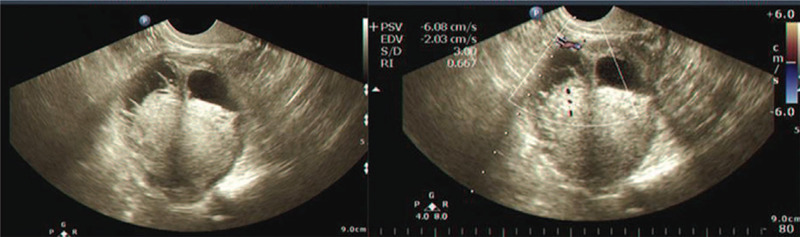
Ultrasound showed that in the left ovarian there was a mass consist of cystic and solid tissue with irregular shape, clear boundaries, and abundant blood flow signal inside.

Ultrasound graph (Fig. 5) found that in the left adnexa region there was a mass consisting of cystic and solid tissues with irregular shape, clear boundary, and abundant blood flow signal. Blood pressure was 153/90 mm Hg, an abnormal T wave with ST slightly shifting down approximately 0.15 mv was detected by ECG and normal structure and function of heart was observed by echocardiograph. The results of tumor markers were normal.

### 5.3 Treatment strategies

**Figure 6:**
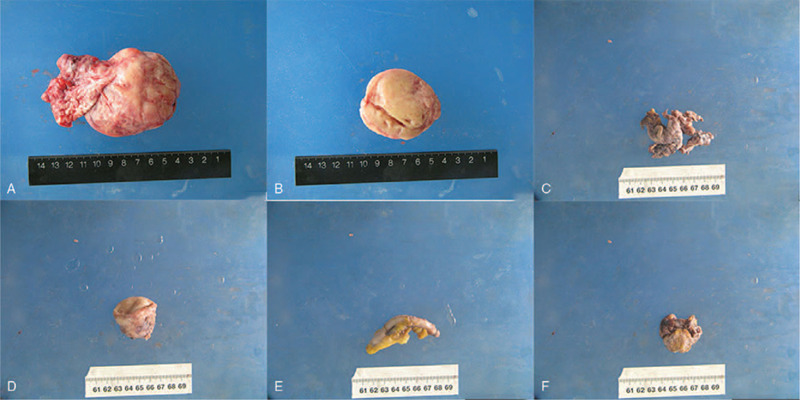
Figure 6 (A-F) showed the tissues resected by laparotomy in case 2.

Cervix resection, right salpingo-oophorectomy, appendectomy, and pelvic lesion resection was conducted (Fig. 6). Tumor metastasis and invasion were found in appendix, left board ligament and infundibulopelvic ligament during exploration intraoperatively. Thus, regular cycles of chemotherapy, combining paclitaxel (Yangzijiang Pharmaceutical Group Co., Ltd. China) with lobaplatin (Hainan Changan International Pharmaceutical Co., Ltd. China), was taken out postoperatively.

### 5.4 Pathological characteristics

**Figure 7:**
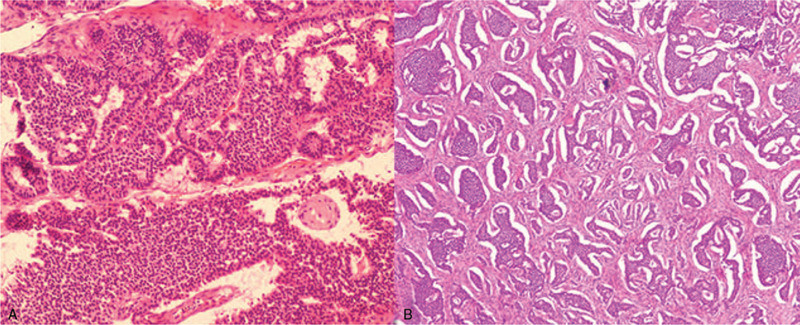
Pathological results showed ovary carcinoid, insular type.

The result of pathological examination (Fig. 7) was ovary carcinoid, insular type. IHC results were shown as follows: CK (AE1/AE3), CgA, Syn, CD56, and CD10 were positive, and calretinin, WT-1, EMA and α-inhibin were negative. Besides, vimentin was partially positive and the positive index of Ki67 was 10%. The patient was diagnosed as POC with stage IIIA according to the 2014 FIGO staging classification for ovarian cancer.^[43]^

### 5.5 Clinical outcomes and follow-up

Multiple metastases, including liver, spleen, retroperitoneum and left iliac bone, were confirmed by abdominal CT scan 5 months postoperatively, thus, we changed paclitaxel (Yangzijiang Pharmaceutical Group Co., Ltd. China) to docetaxel (Jiangsu Hengrui Pharmaceutical Co., Ltd. China). Seven months after surgery, metastases were found in the liver and paraaortic lymph nodes, however, not observed in the spleen. At the last 17-month follow-up visit, the patient felt headache sometimes, constipation occasionally and denied any other discomfort symptoms.

## 6 Discussion

Carcinoid tumor is one of the most prevalent neuroendocrine tumors, and the majority of which are usually observed in gastrointestinal and bronchopulmonary systems, however, with other locations like gynecologic organs relatively rare.[^2^,^3^,^4^] Previous literatures reported that the prevalence of OC was just 0.1% in ovarian neoplasms and 0.8% to 5% in carcinoid tumors.[^7^,^44^] In 1939, Stewart et al first described OC, then Stewart et al and Kurman et al classified it into monodermal ovarian teratomas.[^8^,^45^] To date, the symptom of carcinoid heart disease was occasionally reported, furthermore, diarrhea, and constipation were rarely reported. Therefore, we present 2 rare cases in our hospital of POC tumor, causing symptoms of the diarrhea, constipation, and carcinoid heart disease. Besides, we also reviewed related literatures from 2009 to 2019.

Regarding the origin of OC, currently, it is still unclear. Vora et al^[46]^ suspected it was aroused from neural crest. Niu et al^[47]^ revealed that the insular and mucinous types were considered as midgut derivation, and trabecular and stromal carcinoid were defined as foregut or hindgut derivations.

Considering clinical manifestations, most patients are perimenopausal or postmenopausal females aged from 14 to 83 years.^[48]^ Clinical symptoms of POC are usually not specific, and occasionally abdominal pain, vaginal bleeding, and dysmenorrheal were reported.^[48]^ Besides, rare symptoms like heart disease, diarrhea, constipation, hypoglycemia, and hirsutism have been reported in some cases.[^3^,^7^,^49^]

It is difficult to make accurate diagnosis of OC preoperatively. Ge et al^[50]^ described that the diagnosis and differential diagnosis largely relied on the histopathologic characteristics and the immuno-phenotype. De et al.^[51]^ demonstrated that the diameter of OC ranged from 4 to 25 cm in clinicopathological specimens and Davis et al^[52]^ claimed that the neuroendocrine granules were often found in the plasma of tumor cells under microscope. Electron microscopy could facilitate the identification of these tumors by detecting typical cytoplasmic granules. IHC analysis,[^46^,^49^] such as Syn, CgA, CD56, PYY, and thyroglobulin, could also promote the diagnosis of POC, while in our cases, both tumor tissues were positive for Syn, CgA, and CD56. The specificity of CgA and 5-HIAA was 86% and 35%, respectively.^[53]^ Besides, there was study reporting that the sensitivity of CgA was associated with the disease severity.^[49]^ Recently, Zhang et al^[54]^ reported that there was a close correlation between Ki67 index and patient survival time, and a higher Ki67 index in metastatic carcinoid indicated a worse prognosis when compared with POC. In our study, the Ki67 positive index of case 1 and case 2 was 5% and 10%, respectively. Metastatic carcinoid was observed in case 2, and our results are consistent with Zhang et al.^[54]^

The treatment strategy of POC should take its stage, histology type, patient age, and fertility needs into consideration.^[55]^ For young females in early stage (stage I) who have fertility expectation, fertility sparing surgery could be preferred. For patients in late stage (stage II to IV), comprehensive staging and cytoreductive surgery is of recommendation. For patients with mucinous type of POC, omentectomy and para-aortic lymphadenectomy may be necessary. For insular and trabecular types, TAH+BSO should be selected. In addition, the recurrent and metastatic diseases are usually managed by secondary surgical resection, chemotherapy, and molecular therapy. Chemotherapy could be used in late stage OC.^[56]^ In our study, Case 1 and Case 2 were classified as stage I and stage III, respectively, without and with chemotherapy conducted postoperatively. Satisfactory results were achieved in both patients during follow-up visit. Whether radiation therapy, hormonal therapy, and molecular therapy are useful or not has not been validated. Recently, some molecular medications were reported to help survival in gastrointestinal and pancreatic carcinoids, and is also proved to be useful in OC.[^3^,^56^]

The prognosis of POC is extraordinarily good in early stage, however, there also remains malignant potential,^[44]^ therefore, patients should have regular follow-ups, particular, in patients who underwent fertility sparing surgery. Prognosis is influenced by pathological stage, histological subtype, pathologic components, and proliferation activity. The 10-year survival rate in stage I POC patients is as high as 100%, whereas the 5-year survival rate in later stage decreases to 33%.^[57]^ Authors reported that the prognosis was good in insular, trabecular, and stromal carcinoids of POC when compared with mucinous or undifferentiated type.[^46^,^58^] In our study, both patients here were insular type and exhibited good prognosis, up to now, they achieved satisfactory therapeutic effects without deterioration of general condition.

## 7 Conclusion

POC is an extraordinarily rare disease, and usually with a satisfactory outcome. TAH+BSO with or without postoperative chemotherapy are acceptable treatment choices for primary ovarian carcinoid patients.

## Author contributions

**Conceptualization:** Li-Rong Zhai, Manhua Cui.

**Data curation:** Li-Rong Zhai, Dong-Wei Huang.

**Formal analysis:** Xi-Wen Zhang, Tong Yu, Zhen-De Jiang.

**Funding acquisition:** Yan Jia.

**Investigation:** Yan Jia.

**Methodology:** Xi-Wen Zhang, Tong Yu, Zhen-De Jiang, Dong-Wei Huang.

**Resources:** Tong Yu, Zhen-De Jiang, Dong-Wei Huang.

**Supervision:** Yan Jia, Manhua Cui.

**Visualization:** Tong Yu.

**Writing – original draft:** Li-Rong Zhai, Xi-Wen Zhang, Tong Yu.

**Writing – review & editing:** Yan Jia, Manhua Cui.
